# Recurrent vitreous hemorrhage associated with regressed retinopathy of prematurity in a 47-year-old patient: a case report

**DOI:** 10.1186/1752-1947-8-183

**Published:** 2014-06-10

**Authors:** Masayuki Takeyama, Masayoshi Iwaki, Masahiro Zako

**Affiliations:** 1 Department of Ophthalmology, Aichi Medical University, Nagakute, Aichi 480-1195, Japan

**Keywords:** Vitreous hemorrhage, Retinopathy of prematurity, Vitrectomy

## Abstract

**Introduction:**

Vitreous hemorrhage associated with retinopathy of prematurity is often seen in childhood, but adult onset without retinal break is rare. We describe a case of recurrent vitreous hemorrhage associated with regressed retinopathy of prematurity in a 47-year-old patient.

**Case presentation:**

A 47-year-old Japanese woman with a history of retinopathy of prematurity presented with a visual disturbance in her left eye due to vitreous hemorrhage. Because the vitreous hemorrhage was recurrent and refractory, we performed pars plana vitrectomy combined with lens extraction by phacoemulsification and intraocular lens implantation. No retinal break or retinal detachment was found. No vitreous hemorrhage or other complication occurred in the first six months after surgery.

**Conclusions:**

Vitrectomy, potentially in combination with lens extraction, should be considered in adult-onset recurrent vitreous hemorrhage associated with retinopathy of prematurity.

## Introduction

Vitreous hemorrhage (VH) associated with retinopathy of prematurity (ROP) is often seen in childhood, but adult onset without retinal break is rare [1]. Here, we report a case of recurrent VH associated with regressed ROP without retinal break and retinal detachment in a 47-year-old patient.

## Case presentation

A healthy 47-year-old Japanese woman presented at our hospital complaining of sudden visual deterioration in her left eye. Her best-corrected visual acuity (BCVA) was hand motion in her left eye and 0.5 in her right eye. She had a history of ROP (body weight at birth: 800g), but other details were unclear. Our patient was born in 1965, when there was no consideration of treatment for her ROP.

**Figure 1:**
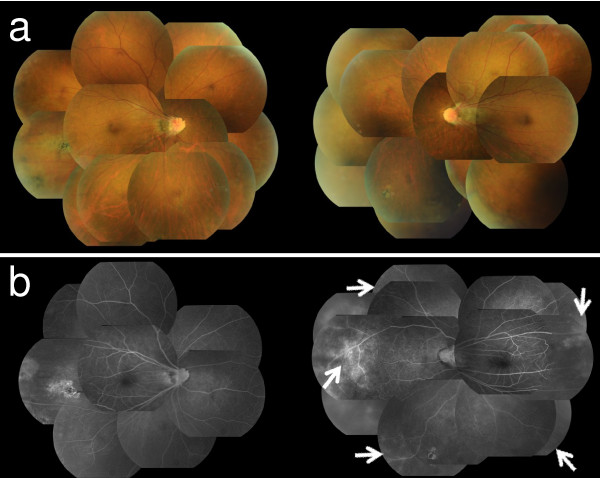
**Regressed retinopathy of prematurity found in both eyes. (a)** Fundus photographs of both eyes taken after the disappearance of the first episode of vitreous hemorrhage in the left eye. Regressed retinopathy of prematurity was found in both eyes. **(b)** Fluorescein angiography taken on the same day. In the left eye, increasing vascular permeability was found in the midperipheral and peripheral retina (*white arrows*), but no neovascularization was observed. In the right eye, regressed retinopathy of prematurity was found, but it was stable.

**Figure 2:**
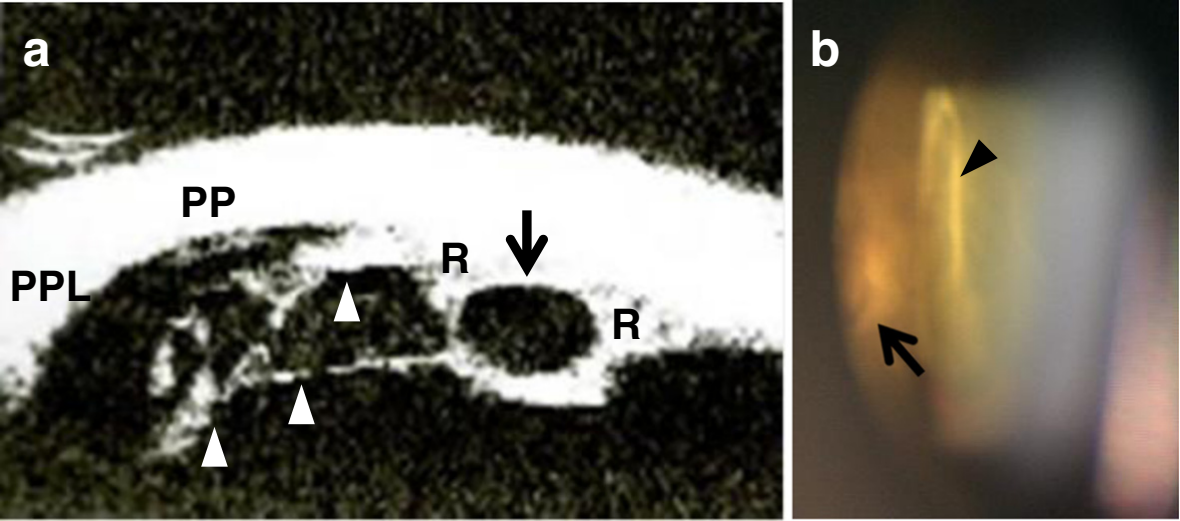
**Membranous tissue and exudative retinal cyst found in the left eye. (a)** Ultrasound biomicroscopy showed membranous tissue adhering to the temporal peripheral retina in the left eye (*arrowheads*). A retinal cyst was detected (*arrow*). **(b)** Slit-lamp photograph taken through Goldmann's three-mirror contact glass. An exudative retinal cyst (*arrow*) and membranous tissue adhesion on the posterior lens capsule (*arrowhead*) are shown. R, retina; PP, pars plana; PPL, pars plicata.

The fundus in her left eye was invisible due to massive VH, but B-scan ultrasound ruled out retinal detachment. Regressed ROP, straightening vessels, pigmentation in the midperipheral retina, and ectopia of a hypoplastic macula were found in her right eye.In her left eye, the VH decreased three weeks later and the BCVA improved to 0.4. The fundus photograph taken after the disappearance of the VH is shown in Figure 1a. Regressed ROP similar to her right eye was found in her left eye. The membranous tissue connecting the temporal peripheral retina and posterior lens capsule, as well as a retinal cystic change, were shown by ultrasound biomicroscopy (Figure 2a). Membranous tissue of adhesion was found at the posterior lens capsule and retinal exudate, corresponding to the retinal cystic lesion on ultrasound biomicroscopy (Figure 2b). Fluorescein angiography in her left eye did not reveal neovascularization, but increased vascular permeability was observed at the midperipheral and peripheral retina (Figure 1b). In her right eye, no neovascularization or increased vascular permeability was detected.

**Figure 3:**
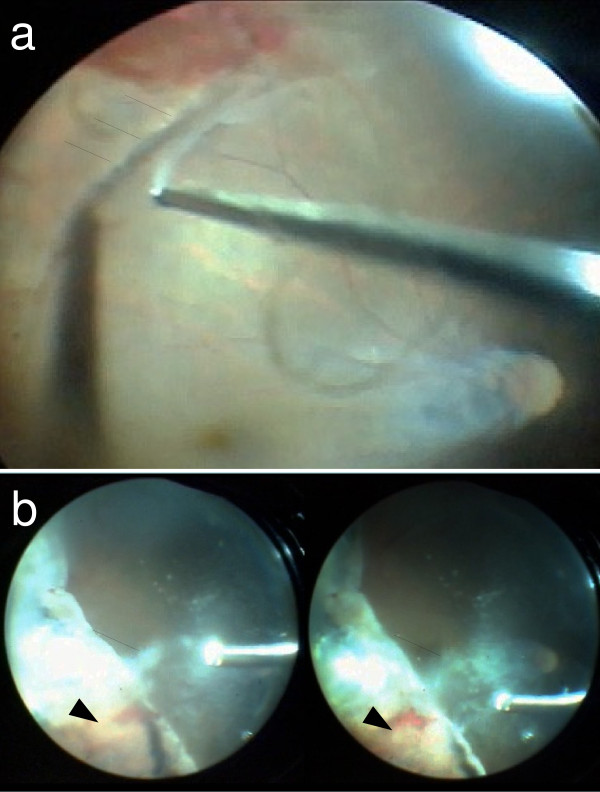
**Findings in the left eye during vitrectomy. (a)** The vitreoretinal adhesion was very tough from the midperipheral to the peripheral area. We could not complete posterior vitreous detachment to the periphery beyond this line (*arrows*). **(b)** With indentation of the sclera, the membranous tissue (*arrows*) and exudative retinal cyst (*arrowheads*) appeared.

**Figure 4:**
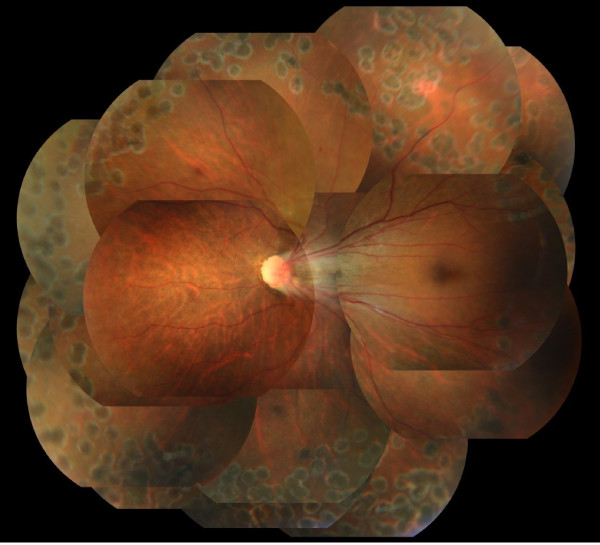
**Fundus photograph of the left eye six months after the surgery.** No vitreous hemorrhage or other complications occurred.

For the next two months, VH recurred twice in her left eye. The left BCVA deteriorated to hand motion after the third episode of VH, and the fundus became invisible. As the VH was refractory, we performed pars plana vitrectomy combined with lens extraction by phacoemulsification and artificial intraocular lens (IOL) implantation four months after her first visit.After lens extraction by phacoemulsification and IOL implantation, core and peripheral vitrectomy were performed. We could have performed posterior vitreous detachment at the posterior pole to the midperipheral area, but the vitreoretinal adhesion was very tough from the midperipheral to the peripheral area. Because posterior vitreous detachment from the midperipheral to the peripheral area could not be completed (Figure 3a), we shaved the vitreous body as much as possible. The area was concurrent with the increased vascular permeability shown by fluorescein angiography (Figure 1b). Via indentation of the sclera, the membranous tissue and exudative retinal cyst were observed (Figure 3b). We excised peripheral vitreous and membranous tissue using a vitreous cutter, and we performed cryocoagulation and laser photocoagulation in the area of increased vascular permeability shown by fluorescein angiography. Neither retinal break nor retinal detachment was found.Six months after the surgery, VH and other complications had not occurred, and the left fundus was stable (Figure 4). The final BCVA in her left eye was 0.6.

## Discussion

The mechanism of VH associated with regressed ROP has been discussed previously. Ruth *et al*. described 14 cases, and the age at late VH ranged from 10 months to 15 years [2]. Ruth *et al*. proposed two mechanisms: abnormal vitreoretinal traction disrupts otherwise normal retinal vessels or vitreous traction disturbs residual cicatricial tissue at the vitreoretinal interface. Our case may correspond to the second mechanism.

The membranous tissue may also play a key role in recurrent VH. Breakage of blood vessels in the membranous tissue during the period of active ROP, with additional deterioration during ageing, may induce recurrent VH. We excised the membranous tissue by vitrectomy and performed both laser photocoagulation and cryocoagulation, which appeared to be effective. Thus, surgical treatment should be considered in such cases of adult-onset recurrent VH.

Though the fundus of her right eye was similar to her left eye, no serious disorders have been observed in her right eye during the follow-up period. Fluorescein angiography demonstrated that the ROP in her right eye is completely silent. The subtle differences in the ROP condition in childhood may have led to the different prognoses in both eyes.

## Conclusions

Adult-onset recurrent VH associated with ROP without retinal break or retinal detachment is rare. Here, we performed pars plana vitrectomy combined with lens extraction by phacoemulsification and IOL implantation. After the surgery, no VH or other complications occurred. According to our experience with this case, we conclude that vitrectomy, potentially in combination with lens extraction, should be considered in such cases.

## Consent

Written informed consent was obtained from the patient for publication of this case report and any accompanying images. A copy of the written consent is available for review by the Editor-in-Chief of this journal.

## Abbreviations

BCVA: Best-corrected visual acuity; IOL: Intraocular lens; ROP: Retinopathy of prematurity; VH: Vitreous hemorrhage.

## Competing interests

The authors declare that they have no competing interests.

## Authors’ contributions

MT and MI analyzed and interpreted the patient data. MZ was a major contributor to writing the manuscript. All authors read and approved the final manuscript.
